# Community-level assessment of dental plaque bacteria susceptibility to triclosan over 19 years

**DOI:** 10.1186/1472-6831-14-61

**Published:** 2014-06-02

**Authors:** Violet I Haraszthy, Prem K Sreenivasan, Joseph J Zambon

**Affiliations:** 1 School of Dental Medicine, University at Buffalo, Squire Hall, 3435 Main Street, Buffalo NY 14214-3008, USA; 2 Colgate-Palmolive Technology Center, 909 River Road, Piscataway NJ 08855, USA; 3 University at Buffalo, School of Dental Medicine, 222 Foster Hall, 3435 Main Street, Buffalo NY 14214-3008, USA

**Keywords:** Triclosan, Antimicrobial susceptibility, Bacteria, Toothpaste, Antimicrobial resistance, Dental plaque

## Abstract

**Background:**

Triclosan is a broad-spectrum antimicrobial agent used in toothpaste to reduce dental plaque, gingivitis and oral malodor. This community-level assessment evaluated the susceptibility of dental plaque bacteria to triclosan in samples collected over 19 years.

**Methods:**

A total of 155 dental plaque samples were collected at eleven different times over 19 years from 58 adults using 0.3% triclosan, 2% copolymer, 0.243% sodium fluoride toothpaste and from 97 adults using toothpaste without triclosan. These included samples from 21 subjects who used triclosan toothpaste for at least five years and samples from 20 control subjects. The samples were cultured on media containing 0, 7.5 or 25 μg/ml triclosan. Descriptive statistics and p values were computed and a linear regression model and the runs test were used to examine susceptibility over time.

**Results:**

Growth inhibition averaged 99.451% (91.209 - 99.830%) on media containing 7.5 μg/ml triclosan and 99.989% (99.670 - 100%) on media containing 25 μg/ml triclosan. There was no change in microbial susceptibility to triclosan over time discernible by regression analysis or the runs test in plaque samples taken over 19 years including samples from subjects using a triclosan-containing dentifrice for at least five years.

**Conclusions:**

This community-level assessment of microbial susceptibility to triclosan among supragingival plaque bacteria is consistent with the long-term safety of a 0.3% triclosan, 2% copolymer, 0.243% sodium fluoride dentifrice.

## Background

Triclosan is a broad-spectrum antimicrobial agent developed over 40 years ago [1] that is added to toothpaste to reduce dental plaque, gingivitis and oral malodor beyond that achieved using toothpastes without triclosan [2,3]. Deasy et al. [4] reported that compared to a fluoride dentifrice, a dentifrice containing 0.3% triclosan, 2% copolymer and 0.243% sodium fluoride reduced dental plaque by an additional 19% and reduced gingivitis by an additional 17% after three months of use, and by an additional 32% and 25%, respectively, after six months. Meta-analysis of sixteen clinical trials [2,5] found that a 0.3% triclosan, 2% copolymer and 0.243% sodium fluoride dentifrice reduced dental plaque by 23%, gingival inflammation by 23% and gingival bleeding by 49% compared to a fluoride dentifrice. In addition to its efficacy in reducing dental plaque and gingivitis more than that achieved with a fluoride dentifrice, triclosan dentifrice has been reported to slow the progression of chronic periodontitis [6] and to prevent recurrent periodontitis [7].

Several studies have examined the effects of the 0.3% triclosan, 2% copolymer and 0.243% sodium fluoride dentifrice on dental plaque bacteria [8-13] and did not find the emergence of triclosan resistant bacteria in oral samples. A recently published study reported that triclosan-resistant bacteria did not develop after five years use of a triclosan-containing toothpaste [14]. The present study examined supragingival dental plaque bacteria in samples collected over nineteen years for changes in susceptibility to triclosan.

## Methods

### Subjects and samples

One hundred fifty-five supragingival plaque samples were collected at eleven different times over 19 years from 58 adults who used a 0.3% triclosan, 2% copolymer and 0.243% sodium fluoride dentifrice (Colgate Total) and from 97 adults who used a dentifrice without triclosan-copolymer (Table 1). In addition, the susceptibility of supragingival dental plaque collected from 21 subjects who used the triclosan dentifrice for at least five years and as long as 13 years was compared to 20 subjects who used a dentifrice without triclosan. All the adults were 18 years of age and older. The University at Buffalo, Health Sciences Institutional Review Board, approved this study.

**Table 1:** Sample years and sample numbers

**Year**	**Number samples**	**No. samples triclosan dentifrice-using subjects**	**No. samples non-triclosan dentifrice-using subjects**
1991	10	5	5
1999	15	5	10
2000	15	5	10
2001	15	5	10
2002	10	5	5
2003	20	6	14
2004	20	6	14
2005	10	5	5
2007	10	5	5
2008	10	5	5
2010	20	6	14
Total	155	58 (37.419%)	97 (62.580%)

### Sample collection and processing

Each sample contained supragingival dental plaque collected from the entire dentition. Teeth were isolated with cotton rolls, air dried, and supragingival dental plaque was collected using a sterile periodontal curette into a single 3 ml tube of pre-reduced anaerobically sterilized Ringer’s solutions. The plaque samples were dispersed by mild sonication and distributed onto media using a spiral plater (Model D2, Spiral Systems, Cincinnati, OH). The media consisted of tryptic soy agar supplemented with 5% defibrinated rabbit blood, 5.0 μg/ml hemin, and 0.5 μg/ml vitamin K_1_. As in previous studies [11,15], triclosan was added to the media at concentrations of 7.5 μg/ml and 25 μg/ml. The concentrations of triclosan used in this study were selected based on the MIC’s for strains of oral bacteria previously described [15]. The 7.5 μg/ml triclosan concentration is equivalent to the MIC for 80% of test strains while the 25 μg/ml triclosan concentration is equivalent to the MIC for 95% of the bacteria [15]. All inoculated plates were incubated under anaerobic conditions (95% N_2_, 10% H_2_, 5% CO_2_) at 35-37˚C. After 5–7 days incubation, total viable colony counts (colony forming units (CFU)/ml) were enumerated using a counting grid (Spiral Systems, USA) on the media without triclosan and on the media containing either 7.5 μg/ml or 25 μg/ml triclosan.

### Statistical analysis

Statistical analysis examined triclosan susceptibility over 19 years. For each evaluation year, an ANOVA compared cultivable bacteria on triclosan-free or triclosan-containing media to determine statistical differences between the numbers of cultivable organisms recovered on both types of media. A linear regression model and the runs (Wald–Wolfowitz) test evaluated trends for microbial susceptibility for each triclosan concentration over time. *P* values less than 0.05 were considered significant. All analyses were conducted using Minitab (State College, PA).

## Results and discussion

Large numbers of bacteria were cultivable on media without triclosan from supragingival dental plaque samples taken from subjects using 0.3% triclosan, 2% copolymer, 0.243% sodium fluoride dentifrice and from supragingival dental plaque samples from subjects using dentifrice without triclosan. Subjects using the triclosan dentifrice demonstrated a mean of 1.36 ×10^8^ CFU/ml while subjects using dentifrice without triclosan demonstrated a mean of 1.6 × 10^8^ CFU/ml. There were fewer bacteria cultivable on media containing triclosan (p < 0.05) than on media without triclosan indicating triclosan inhibition of bacterial growth. Culture of plaque samples collected over nineteen years on media containing 7.5 μg/ml or 25 μg/ml triclosan showed reductions in the mean number of colony forming units of 99.451% (range 91.209 - 99.830%) and 99.989% (range 99.670 -100%), respectively, compared to culture on media without triclosan (Table 2). That is, plaque samples cultured on media containing 7.5 μg/ml triclosan showed an average of 8.8 × 10^5^ CFU/ml while plaque samples cultured on media containing 25 μg/ml triclosan showed an average of 3.2 × 10^4^ CFU/ml. Characterization of dental plaque bacteria cultivable on media containing triclosan [10,11] as well as the triclosan susceptibility of oral and non-oral bacteria [15] has been previously described.

**Table 2:** Susceptibility of supragingival dental plaque bacteria sampled over 19 years

**Statistical parameter**	**7.5 μg/ml**	**25 μg/ml**
Mean	99.451%	99.989%
Standard error	0.092	0.004
Standard deviation	1.145	0.049
Minimum	91.209%	99.670%
Maximum	99.830%	100%
Median	99.701%	100%
Range	8.791%	0.330%

The susceptibility of supragingival dental plaque collected from 21 subjects who used a 0.3% triclosan, 2% copolymer, 0.243% sodium fluoride dentifrice for at least five years and as long as 13 years was compared to supragingival dental plaque collected from 20 subjects who used a dentifrice without triclosan. Supragingival plaque samples from both groups demonstrated susceptibility to both concentrations of triclosan. There was no statistically significant difference in triclosan susceptibility between those who regularly used triclosan dentifrice and those who didn’t (p < 0.05).

Linear regression analysis and the runs test were used to examine antimicrobial susceptibility to triclosan in the supragingival dental plaque samples collected over 19 years. At both 7.5 μg/ml and 25 μg/ml triclosan concentrations, linear regression analyses showed that the slope of a line fitted to the results was not significantly different from zero. This indicates that there were no statistically significant changes in antimicrobial susceptibility discernible over 19 years (p = 0.159 for the 7.5 μg/ml concentration and p = 0.299 for the 25 μg/ml concentration, Table 3). Analyses using the runs test also demonstrated no trends in susceptibility patterns over the course of the study for either triclosan concentration evaluated (p > 0.05).

**Table 3:** Regression analysis for triclosan susceptibility in supragingival plaque samples obtained over 19 years

**Source of variation**	**7.5 μg/ml**	**25 μg/ml**
Model variation. Year to year variation	2.61	0.0003
Residual variation. Unexplained variation ( σ^2^)	1.30	0.379
Total Variation	3.40	0.38
Regression analysis (p value)	0.159	0.299

The 0.3% triclosan, 2% copolymer, 0.243% sodium fluoride dentifrice reduces dental plaque and plaque-associated gingival bleeding around teeth and also around dental implants [16]. Gingival bleeding is a key sign of periodontal disease, especially plaque-associated gingivitis, but gingival bleeding can also be a sign of more serious forms of periodontal disease such as periodontitis. Periodontitis affects over 47% of adults 30 years of age or older in the United States and 64% of adults 65 years and older [17]. Periodontal infections causing periodontitis affect not only the oral cavity but also increases the risk for systemic diseases and conditions such as preterm low birth weight [18], cardiovascular disease [19], diabetes mellitus [20] and chronic obstructive pulmonary disease [21]. By reducing dental plaque and gingivitis, the 0.3% triclosan, 2% copolymer, 0.243% sodium fluoride dentifrice may also reduce periodontal infection-related systemic diseases and conditions.

Triclosan employs several mechanisms to kill bacteria and reduce dental plaque. It kills some bacteria such as *Escherichia coli*[22], *Staphylococcus aureus*[23] and *Bacillus subtilis*[24] by inhibiting NADH- or NADPH-dependent enoyl-acyl carrier protein reductase (*fab*I) [25] during fatty acid synthesis. It also kills bacteria by means of other membranotropic effects [26], by uncoupling oxidative phosphorylation and by inhibiting glycolysis [27].

However, dental plaque infection and bacterial virulence are not the only mechanisms in the pathogenesis of periodontitis. Inflammation plays a significant role. Plaque bacteria initiate an inflammatory cascade leading to chronic inflammation [28]. In addition to its antimicrobial activity, triclosan also has anti-inflammatory properties. Triclosan can suppress microbial pathogen recognition pathway molecules as well as acute and chronic inflammatory mediators [29]. Wallet et al. [30], for example, showed triclosan to be a potent inhibitor of oral epithelial cell LPS-induced pro-inflammatory responses by regulating the TLR-signaling pathway. Further, Mustafa et al. [31] found that triclosan reduced biosynthesis of PGE_2_ by inhibiting mPGES-1 in gingival fibroblasts.

Some bacteria are naturally resistant to triclosan [32] and some laboratory strains can be made resistant. Pseudomonads, for example, exhibit natural resistance to triclosan by means of multidrug efflux pumps [33]. *E. coli* is not naturally resistant but can be made resistant in the laboratory by long term exposure to sub lethal concentrations of triclosan. Induced resistance in the laboratory occurs by increased production of the enoyl-acyl carrier protein reductase or by overexpression of drug efflux pumps [32] or by development of an alternative fatty acid synthesis pathway [34].

In the present study, there was no change in *ex vivo* triclosan susceptibility among supragingival plaque bacteria collected over 19 years and there was no change in *ex vivo* triclosan susceptibility among supragingival plaque bacteria collected from subjects using a triclosan dentifrice for at least five years and as long as 13 years. This study differs from previous reports in that those samples were taken from subjects participating in clinical trials of triclosan-containing dentifrice. In the present study, subjects were neither included nor excluded based on the type of dentifrice, amount of dental plaque, degree of gingival inflammation or oral health status. The subjects in this study used different mouth rinses, chewing gum, and oral hygiene aids and were possibly exposed to other sources of triclosan as in hand soaps and cosmetics. They had different access and utilization of professional oral health care services and exposure to antibiotics or other antimicrobial agents.

Findings in the present community-level assessment are consistent with previous clinical trials (Table 4). Zambon et al*.*[9] examined supragingival dental plaque in 40 adults who used a 0.3% triclosan, 2% copolymer and 0.243% sodium fluoride dentifrice compared to 41 adults using the same dentifrice without triclosan/copolymer. After 28 weeks, there was no difference between groups except for statistically significant reductions in fusiforms, spirochetes and staphylococci and significant increases in *Streptococcus sanguis* in the test group compared to the control group. Jones et al*.*[8] examined interproximal plaque bacteria and salivary *S. mutans* and candida in 13 female adults who used a 0.2% triclosan, 0.5% zinc citrate dentifrice for seven months compared to 13 female adults who used the same dentifrice without 0.20% triclosan, 0.50% zinc citrate. They found no difference between groups in the number of salivary *S. mutans* or candida, no evidence for a change in the number of predominant plaque bacteria i.e. population shifts, nor evidence for an increase in bacterial resistance to triclosan. Walker et al*.*[10] examined 70 subjects using 0.3% triclosan, 2% copolymer and 0.243% sodium fluoride dentifrice twice a day for six months with surveillance for an additional six months. They found no evidence for unfavorable shifts in supragingival plaque bacteria and no correlation between use of the triclosan dentifrice and the number of triclosan-resistant bacteria, the resistant bacterial taxa, or the number of subjects harboring triclosan-resistant bacteria. Zambon et al*.*[11] examined 73 subjects using 0.3% triclosan, 2% copolymer and 0.243% sodium fluoride dentifrice twice a day for six months with surveillance for an additional six months. Both test and control groups (71 subjects) harbored triclosan resistant supragingival plaque bacteria, mainly *Veillonella dispar*. There was no evidence for unfavorable shifts in supragingival plaque bacteria from use of the triclosan dentifrice and no evidence for the development of triclosan resistance. Fine et al*.*[12] examined 71 subjects using a 0.3% triclosan, 2% copolymer and 0.243% sodium fluoride dentifrice for six months and for an additional six months post-treatment. There were no detrimental shifts in supragingival plaque bacteria and no overgrowth in opportunists, periodontal pathogens, or cariogenic bacteria in either test or control. There was no increase in the proportion of supragingival plaque bacteria resistant to triclosan and there was no increase in the minimum inhibitory concentrations for triclosan against *Actinomyces viscosus* or *Veillonella parvula* in test or control groups. Sullivan et al*.*[13] examined the *in vitro* sensitivity of triclosan against viridans streptococci and normal oral flora in saliva samples obtained from 9 adults who used a 0.3% triclosan, 2% copolymer and 0.243% sodium fluoride dentifrice twice a day for two weeks. No major changes were detected in the normal oral flora and there were no differences in susceptibility to triclosan, benzyl penicillin, gentamicin, erythromycin, tetracycline or fusidic acid between streptococci obtained pre- or post-treatment.

**Table 4:** Studies of the effect of triclosan dentifrice on oral microorganisms

**Study**	**Test dentifrice formulation**	**Subjects**	**Study length**	**Methods**	**Results**
Jones et al*.*[8]	0.2% triclosan	• Test = 13 adult females	• 4 mon. washout	• Interproximal plaque and saliva samples	• No detectable shifts in plaque oral ecology
	0.5% zinc citrate	• Control = 13 adult females	• 7 mon. use	• Bacterial culture	• No difference between groups in numbers of *S. mutans* or candida
		• 20–50 years of age		• Salivary *S. mutans* and candida enumerated	• No evidence of increased bacterial resistance to triclosan
				• MIC’s by agar dilution	
Zambon et al*.*[9]	0.3% triclosan	• Test = 40 adults	• 6 mon. use	• Supragingival plaque samples	• No unfavorable shifts in plaque bacteria
	2% copolymer 0.243% Na fluoride	• Control = 41 adults	• 6 mon. post-use	• Bacterial culture	• Significant reductions in fusiforms, spirochetes and staphylococci and increases in *S. sanguis* in triclosan group
				• Phase contrast microscopy	
				• Immunofluorescence microscopy	
				• MIC’s by agar dilution	
Walker et al*.*[10]	0.3% triclosan	• Test = 70 adults	• 6 mon. use	• Supragingival plaque samples	• No detrimental shifts in plaque bacteria
	2% copolymer 0.243% Na fluoride	• Control = 74 adults	• 6 mon. post-use	• Bacterial culture	• No correlation between triclosan dentifrice and number of triclosan-resistant bacteria, resistant bacterial taxa, or number of subjects harboring tricosan-resistant bacteria
				• Phase contrast microscopy	
				• Immunofluorescence microscopy	
				• MIC’s by agar dilution	
Zambon et al*.*[11]	0.3% triclosan	• Test = 73 adults	• 6 mon. use	• Supragingival plaque samples	• No unfavorable shifts in plaque bacteria
	2% copolymer 0.243% Na fluoride	• Controls = 71 adults	• 6 mon. post-use	• Bacterial culture	• Higher levels of fusiforms in test group
				• Phase contrast microscopy	• Higher levels of neisseria and *P. gingivalis*-infected
				• Immunofluorescence microscopy	subjects in controls
				• MIC’s by agar dilution	• Both test and controls subjects exhibited triclosan resistant bacteria
					• No evidence for development of triclosan resistance
Rosling et al*.*[7]	0.3% triclosan	• Test = 20 adults	• 36 mon.	• Subgingival plaque samples	• No detrimental shifts in plaque bacteria
	2% copolymer 0.243% Na fluoride	• Control = 20 adults		• Bacterial culture	• Lower total viable bacterial counts in triclosan group compared to control
					• No changes in gingival health-associated bacteria
Fine et al*.*[12]	0.3% triclosan	• 68 adults	• 6 mon. use	• Supragingival plaque samples	• No detrimental shifts in plaque bacteria
	2% copolymer 0.243% Na fluoride		• 6 mon. post-use	• Bacterial culture	• Decreased spirochetes in triclosan group
				• Darkfield microscopy	• No increased proportion of triclosan resistant bacteria
				• Immunofluorescence microscopy	• No increase in triclosan MIC’s for *Actinomyces viscosus* or *Veillonella parvula*
				• MIC’s by agar dilution	
Cullinan et al*.*[6]	0.3% triclosan	• Test = 179 adults	• 5 years	• Subgingival plaque samples	• No overgrowth of *A. actinomycetemcomitans*, *P. intermedia*, or *P. gingivalis*
	2% copolymer 0.243% Na fluoride	• Control = 178 adults		• Enzyme-linked immunosorbent assay	
Sullivan et al*.*[13]	0.3% triclosan	• 9 adults	• 2 weeks	• Saliva samples	• No major changes in normal oral flora
	2% copolymer 0.243% Na fluoride			• Bacterial culture	• No change in streptococcal susceptibility to triclosan, benzyl-penicillin, gentamycin, erythromycin, tetracycline or fusidic acid
				• MIC’s by agar dilution	
Cullinan et al*.*[14]	0.3% triclosan	• Test = 18 adults	• 5 years	• Supra and subgingival interproximal plaque samples	• Triclosan MIC’s similar for isolates from both groups
	2% copolymer 0.243% Na fluoride	• Control = 22 adults		• Bacterial culture	• Triclosan dentifrice does not lead to increased MIC’s for oral bacteria
				• MIC’s by agar dilution	
				• Isolates identified by 16S rDNA sequencing	

In contrast to supragingival plaque, two studies reported the effect of a triclosan dentifrice on subgingival dental plaque. Rosling et al*.*[7] examined 20 test subjects and 20 control subjects over 36 months and found lower numbers of subgingival bacteria and lower numbers of subjects harboring periodontal pathogens in the triclosan dentifrice group compared to the control group. Cullinan et al*.*[6] used enzyme-linked immunosorbent assays for *Actinobacillus* (now *Aggregatibacter*) *actinomycetemcomitans*, *Prevotella intermedia*, and *Porphyromonas gingivalis* and found no overgrowth of these species in 179 adults using 0.3% triclosan, 2% copolymer, 0.243% sodium fluoride dentifrice for five years compared to 178 control subjects.

A recent study by Cullinan et al*.*[14] examined supra and subgingival interproximal dental plaque samples from 18 subjects using a 0.3% triclosan, 2% copolymer, 0.243% sodium fluoride dentifrice for 5 years and 22 subjects using a control dentifrice without triclosan/copolymer. Like the present study, 5 years use of the triclosan dentifrice did not increase triclosan minimum inhibitory concentrations for oral bacteria and did not lead to triclosan-resistant oral bacteria. The minimum inhibitory concentration for oral bacterial isolates in their study from both their triclosan and control groups ranged from 12.50–50 μg/mL, consistent with the mean growth inhibition of 99.451% and 99.989% on media containing 7.5 μg/ml and 25 μg/ml triclosan, respectively, found in the present study. The majority of species cultivable at lower triclosan concentrations in their study were common to both groups and included *Veillonella parvula* and *Campylobacter gracilis* isolates which had the highest MICs. This is consistent with a previous study that also found that triclosan-resistant species were similar between triclosan and control groups and were predominantly *V. dispar*[11].

## Conclusions

There were no changes in antimicrobial susceptibility in dental plaque samples taken over 19 years including samples taken from subjects who used a 0.3% triclosan, 2% copolymer, 0.243% sodium fluoride dentifrice for at least five years.

## Competing interests

Prem K. Sreenivasan is an employee of the Colgate Palmolive Company that is paying article-processing charges for this paper. Violet Haraszthy and Joseph Zambon declare that they have no competing interests.

## Authors’ contributions

All authors contributed to the conception and design of the study. VH collected samples and supervised the clinical laboratory assays. JZ collected samples and drafted the paper. PS performed the statistical analysis. All authors read and approved the final manuscript.

## Pre-publication history

The pre-publication history for this paper can be accessed here:

http://www.biomedcentral.com/1472-6831/14/61/prepub
